# News and Notes

**Published:** 1899-07

**Authors:** 


THE Professor of Hygiene at Konigsberg, Erwin v. Esmarch, has accepted a call to Göttingen in the same capacity.

---

MR. LAWSON TAIT, of Birmingham, England, one of the most brilliant abdominal surgeons of the age, recently died in that city at the age of fifty-one. He had many carping critics, but all recognized his wonderful ability.

---

MR. IRA DE LAMETER, formerly with C. O. Tyner, and Mr. Peyton H. Todd, formerly with Lamar Drug Co., have opened a first-class new drug store, corner Marietta and Forsyth Sts., under firm name of De Lameter & Todd.

---

DR. MAX SANGER, the noted gynecologist of Leipsic, has accepted a call to the German University of Prague, as professor of obstetrics and gynecology in the place of Professor Rosthorn who was recently transferred to Innsbruck. Dr. Sanger was Privat-Dosent in Leipzig for a number of years.

---

THE WILLIAM F. JENKS MEMORIAL PRIZE.—The fifth triennial prize of five hundred dollars, under the deed of trust of Mrs. William F. Jenks, will be awarded to the author of the best essay on "The Various Manifestations of Lithemia in Infancy and Childhood, with the Etiology and Treatment." The conditions annexed by the founder of this prize are that "prize or award must always be for some subject connected with obstetrics, or the diseases of women, or the diseases of children"; and that "the trustees under this deed for the time being can, in their discretion, publish the successful essay, or any paper written upon any subject for which they may offer a reward, provided the income in their hands may, in their judgment, be sufficient for that purpose, and the essay or paper be considered by them worthy of publication. If published, the distribution of said essay shall be entirely under the control of said trustees. In case they do not publish the said essay or paper, it shall be the property of the College of Physicians of Philadelphia." The prize is open for competition to the whole world, but the essay must be the production of a single person. The essay, which must be written in the English language, or if in a foreign language, accompanied by an English translation, must be sent to the College of Physicians of Philadelphia, Pennsylvania, U. S. A., before January 1, 1901, addressed to Richard C. Norris, M.D., chairman of the William F. Jenks Prize Committee. Each essay must be typewritten, distinguished by a motto, and accompanied by a sealed envelope bearing the same motto and containing the name and address of the writer. No envelope will be opened except that which accompanies the successful essay. The committee will return the unsuccessful essays if reclaimed by their respective writers, or their agents, within one year. The committee reserves the right not to make an award if no essay submitted is considered worthy of the prize. James V. Ingham, M.D., Secretary of the Trustees.

